# Social Ecological Dynamics of Catchment Resilience

**DOI:** 10.3390/w13030349

**Published:** 2021-01-30

**Authors:** W. Neil Adger, Katrina Brown, Catherine Butler, Tara Quinn

**Affiliations:** Geography, College of Life and Environmental Sciences, University of Exeter, Exeter EX4 4RJ, UK

**Keywords:** community resilience, social ecological systems, institutional fit, governance, social processes

## Abstract

Catchment resilience is the capacity of a combined social ecological system, comprised of water, land, ecological resources and communities in a river basin, to deal with sudden shocks and gradual changes, and to adapt and self-organize for progressive change and transform itself for sustainability. This paper proposes that analysis of catchments as social ecological systems can provide key insights into how social and ecological dynamics interact and how some of the negative consequences of unsustainable resource use or environmental degradation can be ameliorated. This requires recognition of the potential for community resilience as a core element of catchment resilience, and moves beyond more structural approaches to emphasize social dynamics. The proposals are based on a review of social ecological systems research, on methods for analyzing community resilience, and a review of social science and action research that suggest ways of generating resilience through community engagement. These methods and approaches maximize insights into the social dynamics of catchments as complex adaptive systems to inform science and practice.

## 1 Introduction

Catchment resilience is the dynamic capacity of interacting social and ecological elements of an area bounded by a river basin to cope with disruptions and shocks, and to adapt to and change in new circumstances. The extent of catchment resilience and the processes through which it is generated have been extensively researched, giving the concept meaning and making it observable and measurable. Social analysis of catchment resilience emphasizes process and action, rather than assets or competences, and the metrics to assess catchment resilience are diverse. However, catchment resilience is not only empirically observed, it is also a normative concept – a goal to be desired and enacted, assuming that resilience is universally and always a desirable trait of a system.

This paper conceptualizes catchment resilience as a characteristic of a complex dynamic social ecological system. We review insights from studies and research that examine catchments as social ecological systems; they characterize catchments as involving interactions between human society and the environment. This view emphasizes how rivers, land use, settlements, hazards and institutions interact to produce systems in stable and less stable states. While the original objective of social ecological systems analysis was descriptive, subsequent work aimed to present a more analytical framework, which could also be used for comparative analysis. Anderies, Janssen, and Ostrom [1], for example, developed a simple model to analyze the robustness of social ecological systems which aims to identify the key interactions within systems, recognizing both the designed and self-organized components of a social ecological system and how they interact. Ostrom [2] sets out a generic framework that can be applied and refined by scholars to clarify the structure of a social ecological system to understand how any particular solution might affect management outcomes and sustainability, applied to diverse governance systems and contexts.

The focus in this paper is on the social dynamics of catchments, so differs from a more structural approach. Here we highlight key dimensions including: the role of institutions and decision-making, the role of communities within a catchment, and the potential for trade-offs between community resilience and other dimensions. We explore the role of social science concepts and methods in both describing the state of catchment resilience, and in providing insights into the malleable and fluid nature of catchment resilience. In other words, social research can both advance explanation of catchment resilience and contribute to fostering and building it.

## 2 Key Features of Catchments as Social Ecological Systems

Catchments are social ecological systems in the general sense that elements of the biophysical world affect each other in bounded timescales and spatial scales (e.g. vegetation cover affects hydrology), while social responses and parameters also directly affect parts of the system, through altering land use or other processes [3,4]. Yet social systems are much less geographically bounded and are affected by processes at multiple scales, up to global processes and markets [5,6]. Hence, only examining actions or decisions by social agents on the ground within catchments misses many of the challenges to which social-ecological systems are subject. For example, the decision of multiple independent farmers to plant maize, or plough up hedgerows cannot be explained by contagious behavior or even by current prices of commodities, but rather through understanding diverse economic motivations and social pressures and perceptions of duty and stewardship.

We develop a re-configured social ecological systems perspective that includes the incorporation of the hitherto under-emphasized, and often hidden, social dimensions that determine catchment resilience. This addresses some of the common criticisms of social ecological systems approaches, that they exclude a nuanced and dynamic analysis of social and political aspects [7]. The emphasis here is on attachment to place, identity, and the imperatives that shape how agencies and institutions make decisions in their own interests. These insights are derived from social ecological systems research; from sociological, geographical and psychological insights into place and identity; policy sciences on environmental governance; and political ecology perspectives on the nature of power relations that structure how catchments are managed [8–11].

Diverse evidence shows that people and communities living within catchments clearly cope with and respond to change and to unforeseen but predictable hazards, such as floods and drought. But the evidence also shows that the ability to bounce back from such impacts is highly uneven in society [12]; that lessons are often not learned by individuals or institutions; that responsibilities fall between cracks with institutions not necessarily fit for purpose [13]; and that many impacts in the social realm are hidden, and extend way beyond the forms of economic disruption that tend to dominate analyses [14], and may be temporally and spatially displaced.

Are some of these challenges, revealed by social science and interdisciplinary analyses, easily explained, or amenable to action and intervention? We suggest that understanding catchments as social ecological systems provides key insights into how social and ecological dynamics interact and how some of the negative consequences can be ameliorated to identify sustainable catchment management options. In essence, this requires recognition of the potential for community resilience as a core element of catchment resilience. By studying community resilience, we can gain insights into the social dynamics of catchments as complex adaptive systems to inform science and practice.

For social ecological systems, resilience refers to the magnitude of disturbance that can be absorbed before a system changes to a radically different state. Much systems-oriented research shows that the societal elements fundamentally regulate the extent of that resilience and include dimensions such as, the capacity to self-organize and the capacity for adaptation to emerging circumstances [15]. Recently, emphasis has moved away from persistence to understanding resilience related to adaptability and transformation, recognizing that social ecological systems are often radically changed, particularly as a result of human intervention. The terminology is often difficult to penetrate in these areas because it is used in a scientific and analytical sense on one hand, and as a policy goal on the other. In systems science, elements of emergence, timescale and the likelihood of stability of a system are understood as determinants of resilience of a system as an analytical construct. But in environmental management, resilience is often presented as a normative goal of policy or management to be sought after, rather than as a system property.

The key elements of resilience relating to society are the capacity of people and institutions to adapt, and the feasibility of their doing so within the constraints they face. The most commonly identified elements of robust catchments across all studies, identified in a meta-analysis by Rodina [16] are robustness of systems, and having some buffering or redundancy in the system. The analysis also shows, however, that integrated assessments of catchment resilience highlight social dynamics, particularly social learning and participation, collaboration and local knowledge, as critical elements [14]. Hence current catchment management at least recognizes that social responses are integral, even where agencies are somewhat wary or have less capacity to influence such social processes [17].

A second key element is how resilience is directly beneficial to individuals and collectively to society. These latter elements can be observed and measured in terms of well-being, or in the absence of well-being through stress and health. Hence one set of indicators of catchment resilience may be the health and well-being of the populations living within it. There is significant evidence that where catchments are characterized as non-resilient, when populations are exposed to hazards they cause severe impacts on health and well-being, including burdens of disease, risk of injury, loss of material assets and wellbeing, and mental ill-health [18–21].

Social ecological system resilience rests on robust institutional arrangements and arrangements that recognize vulnerable populations and risks within them. Risks to people and property are unevenly distributed across catchments, with upstream and downstream risks, but also have diverse sets of property rights and responsibilities. Hence a key element in catchment resilience is the distribution of public and private responsibility for risks, as elaborated in the sections below. The public and private mix of responsibilities is not fixed, but rather evolves and is mediated through markets, amongst other things. Flood insurance for example, is often dominated by private sector investments and individuals voluntarily adopting and taking out insurance. But it is underwritten by public investments.

The ability of catchment planning to foster harmonious communities depends then on the acceptability of the burden of risks between the public and private sectors. In many instances, the expectations that communities have for their protection are not met when extreme events occur, leading to crises in the legitimacy of public agencies. Such crises have been documented for unprecedented failures, such as the government response to the Hurricane Katrina disaster in Louisiana in 2005 [22]. One general lesson from these insights is that public expectations and responsibilities themselves are important components of the dynamics of social ecological systems.

## 3 The Relationship between Catchment Resilience and Community Resilience

Understanding social dynamics of catchment resilience might well start with understanding the resilience of communities that live within a catchment. Community resilience is a topic of interest across a range of scientific fields, including community development, social work, disaster studies and so on, as well as being a focus of much policy work around responses to extreme events, such as floods and other emergencies. Catchment-based approaches to resilience in this sense involve drawing on, developing, and engaging the capacities and capabilities of those that live within its boundaries, but as noted above, resilience will be influenced by social, political, economic, and cultural processes beyond the boundaries of a catchment. Community resilience encompasses a range of aspects and components, emphasized by different sub-fields and sectors, leading to challenges for definition and measurement.

### 3.1 Defining Community and Defining Community Resilience

Community resilience has been broadly defined as ‘a community’s collective capacity to function in, respond to, and potentially influence an environment characterized by continuous change, uncertainty, and crisis’ [23] (p. 24). Similarly, the concept of social resilience highlights the collective and systemic nature of the phenomenon: ‘the ability of communities to withstand external shocks to their social infrastructure’ [24] (p. 361), including both physical, social, and economic shocks. Hence community resilience relates primarily to collective capacity and related processes and social relations, rather than being a sum of attributes of individuals.

But ‘community’ is variously defined and often a ‘slippery’ concept. The term can mean different things to different people at different times: it cannot be simply or satisfactorily defined by location or by attention to networks. Yet at its core, community involves a necessary focus on the spatial and material aspects of members. Communities of locality, communities of interest, and communities of identity are not necessarily found in a single locality [25]. In the context of catchments as social ecological systems, the features of communities of locality are critically important. In their review of community resilience to climate change, Twigger-Ross et al. [26] suggest three elements of community are relevant for resilience: spatial or geographical; social relations and structures; and psychological elements, such as sense of belonging and othering. These locality, interest and identity dimensions of community are the key ways to understand communities in the context of catchment resilience and are clearly manifest at different scales.

### 3.2 Measuring Community Resilience

There is a diversity of approaches in research and practice to identify and work with community resilience. The most common approaches to measurement of community resilience involve indirect proxies, focusing on the presence of resources, capital and competences that build different capacities in communities [27]. Integrative methods used to measure community resilience in this way often involve aggregation of perceptions or capabilities of individuals within communities [28]. However, emerging insights show how the elements of space, interest and identity highlighted above can expand the measurement of community and make it more useful and comprehensive. For example, Norris et al. [29] emphasize the ways that integrated sets of linked capacities, such as social capital and community competence, enable community resilience by merging and rebounding in various ways, rather than operating as separate entities that can be understood as distinct or independent elements.

Hence emerging cross-disciplinary social science analysis of community resilience moves away from the idea that it can be reduced to a simple measure or index, toward recognition of the relational, subjective, cross-scale and dynamic nature of resilience [23,30]. An accompanying shift has seen calls for a need to understand community resilience in the face of multivariate, intersecting, and uncertain risks. In these social ecological systems framings, resilience is treated as an emergent property of community interaction and can only be understood with attention to particular cases in practice [23]. These approaches in effect emphasize the system-level dimensions of resilience, highlighting interactions between scales, capacities, and multiple risks in any given context. This has seen new efforts to characterize different dimensions of community resilience that are most important for understanding such interactions and offer a deeper basis for engagement. Table 1 summarizes consensus on dimensions that are core to community resilience: place attachment; leadership; community cohesion and efficacy; community networks; knowledge and learning [23].

Research in different contexts has shown the importance of these dimensions of community resilience in understanding impacts of external stresses. For example, in an empirical analysis of community recovery after major flood events at two sites in the UK, Quinn et al. [27] have shown that there is a strong relationship between people’s feelings of belonging and connections with their wider community, and their sense of their own well-being. Results such as this suggest the importance of understanding community resilience processes for responses to disasters, as well as attempts to anticipate and prepare for them. The wider literature on community resilience highlights again the centrality of direct forms of engagement with the people living in any given catchment in order to understand emergent dimensions of community resilience, their interactions, and implications for outcomes in terms of health, wellbeing, and more broadly capabilities [31]. Active involvement in communities facilitates cooperative efforts, such as shaping social institutions [32], as well as having a positive impact on wellbeing [33].

But engagement of communities and actions of individuals is limited in promoting overall catchment resilience, when power structures and governance structures constrain actions at different scales. Understanding capacities is not about passive or static traits but about understanding relations and processes. In essence, communities matter and are a key aspect of the social dimensions of catchment resilience, but community resilience does not equate to catchment resilience. Importantly, dynamic linkages and interactions between individuals, households, organizations and institutions at different scales act as constraints and challenges to community resilience.

## 4 Challenges and Constraints to Catchment Resilience

### 4.1 Issues of Scale: Fit, Misfit and Risks

Any systems approach to catchment resilience must be clear on the boundaries of the system and the scale and scope for action. A significant hurdle in the operationalization of catchment management is clearly the diverse actors and jurisdictions that cut across scales. There is, however, significant policy science evidence on decision-making on catchment planning when the boundaries, jurisdictions and temporal scales of decision-making are not aligned. It draws on core concepts of institutional fit and misfit [34–36], and of understanding risks in decision-making processes to demonstrate challenges and limitations of implementing catchment resilience.

Transforming to a catchment-based approach which integrates diverse landscapes, stakeholders and communities is demanding. Whilst it makes scientific sense, it creates a series of challenges for institutions and decision-making. Many of these challenges relate to cross-scale interactions and misfits between the remit or jurisdiction of different agencies, the different sectorial agencies involved in different parts of the catchment system and the different subsystems, outlined in Box 1.

So what does this mean for catchment resilience? Walker et al.’s integrative study of the Goulburn Broken catchment in Australia [38] is one of the first analyses of catchment resilience to examine these scale dynamics. They show that intervening to address any one of these (e.g., financial viability of farms, water extraction or tree cover), or acting at a single scale will have significant knock on effects – or potentially trade-offs – for other parts of the catchment which ultimately mean sustainable management of the catchment overall is likely to fail. Anderies et al. [39] analyzing the same catchment, further explain how failure to account for cross-scale and sub-system interactions, means that sequential management decisions historically have resulted in erosion of resilience of the system. A series of crisis-driven decisions have increased vulnerability, and thereby reduced future options. They identified a pattern they describe as a ‘pathological cycle of resource degradation’, where optimizing for high output from irrigated dairy activities has made the system more vulnerable to shifts in weather and climate, and social and political processes.

Institutional fit is an especially demanding problem in complex social ecological systems such as catchments. Epstein et al. [40] examine the issue of institution fit, delineating three types of problems. *Ecological fit* represents a technical approach focusing on whether institutions match the ecological or biophysical problems they are meant to address. *Social fit* is concerned with congruence between institutions and the preferences, values, and needs of human actors. *Social–ecological system fit* seeks to uncover context-specific institutional arrangements that are likely to contribute to the sustainability of social ecological systems, such as catchments. Bunce et al. [35] demonstrate the problem of misfits through their examples of river basin management in southern Africa, where downstream farmers are negatively impacted by upstream water management, increasing their vulnerability to weather extremes. This results in transferring vulnerability – from one place to another and from one set of actors or stakeholders to another – and also in this case between countries and jurisdictions (South Africa and Mozambique). This is also analyzed for catchments in France, UK and South Africa by Barreteau et al. [41].

Therville et al.’s study of land use planning and coastal management in the Languedoc in southern France provides an example of how mis-coordination between multiple sectors and complex cross-scale interactions results in renewed or emergent fragilities [42]. They outline three challenges in managing dynamic complex landscapes or catchments such as the Languedoc. The first relates to the constraints and opportunities represented by cross-scale implementation challenges. Second are the consequences of implementation on others at different scales and levels (the trade-offs and vulnerability transfers). Third concerns the mismatch that occurs when authority or jurisdiction is not coterminous with either the problem (flood management) or the resource (the river basin). In a complex system such as a large catchment, add the impacts of climate change, changes in demography, and increased urbanization and competition for land, then the tendency to manage day-to-day problems and avoid strategic action is accentuated. In this case, the commitment to catchment resilience must be shared across institutions and decision-makers at all scales and jurisdictions.

The Languedoc study explores the extent to which major shifts – for example relocation of coastal development and new planning controls are enabled and constrained. In the current context of budget cuts which mean authority is transferred to local level, but without corresponding financial support, this might result in greater fragmentation or new partnerships between public, civil society and non-government groups. This shift is documented in the case of the UK by Naylor et al. [43] who show how new partnerships forged by fiscal austerity amplify certain risks for different policy actors.

### 4.2 Second Order Risks and Challenges to Management Institutions

Even where institutions can be aligned in terms of spatial scale, the processes of decision-making within them can act against catchment resilience. But why do institutions not act for long term resilience? Analyses from political science that examine the internal dynamics of organizations show that the continuity and reputation of the organisations themselves is foremost and often dominant in decision-making [13,44]. So rather than make decisions solely on the basis of external perturbations to catchments, responsible organizations are enthralled to so-called second order risks to their own legitimacy and continued operation.

The role of second order risks in decision-making on resilience has been examined for the case of Cornwall in UK, and in response to severe winter storms from 2013 and 2014. Second order risks—particularly reputational risks—were found to influence decisions and to prompt actions [43]. First order risks refer to both the physical risks to society such as flooding or storm events, and the explicit societal obligation or responsibility of an organization or individual to reduce uncertainty or harm—for example building flood defenses. Second order risks refer to the risks to the organization relating to legitimacy and blame, namely reputation management, that the individual and organization need to manage in order to maintain the successful continuation of the organization. With increased public accountability managers are increasingly integrating second order risk concerns into their decision-making processes and this might militate against innovation or adaptive management, when second order risks require particular responses or defensive actions, for example focusing on predictive statistics rather than probabilistic approaches in order to manage interactions with the public. In Cornwall building back existing coastal defenses went against longer-term coastal management strategy (articulated through Shoreline Management Plans) but were instigated as a form of crisis-management response during the 2013 and 2014 period of winter storms.

### 4.3 Evolving Multi-Level and Polycentric Solutions

Multi-level and polycentric governance are often suggested as a means of managing complex cross-scale and multiple use systems such as catchments. Morrison’s review [37] shows how polycentric governance has increasingly gained traction among both scholars and policymakers. Polycentrism is a model of governance that actively steers local, regional, national, and international actors and instigates learning from experience across multiple actors, levels of decision-making, and temporal scales. A polycentric system is made up of many autonomous units that are formally independent of one another but which choose to act in ways that take account of others through self-organized processes of cooperation and conflict resolution. But these governance systems are not without problems, especially in terms of power and access to decision-making by different actors within the governance system.

Pahl Wostl et al.’s comparative study of catchment resilience in integrated flood management [45] provides evidence that effective implementation is a multilevel process that cannot be prescribed from the top nor driven from the bottom only. A balance is required which fluctuates, meaning that over time, one or the other direction of influence may dominate. Long-term sustainability depends on the effectiveness of the links between informal settings and formal policy processes. Informal spaces are important to support the integration of knowledge and experimentation with innovative approaches. Vertical integration is important to involve actors from the implementation level in policy development and to support feedback experiences from implementation to strategic goal setting and policy formulation.

## 5 Methods, Metrics and Action for Catchments Resilience

Exploration, identification and measurement of catchment resilience requires a range of methods and metrics. Traditionally, a focus of catchment resilience research has been the measurement of stocks and flows of biological, hydrological and environmental components to identify potential strengths and vulnerabilities of catchments to shifts in social and ecological processes. The focus is on keeping catchments functioning similarly to their existing regime and the measurements of stocks and flows inform modelling of catchment dynamics to analyze susceptibility to change [46]. These studies set out to model the interactions and the metrics used including components such as precipitation and discharge, daily temperatures, which are either measured in the field or from already modelled data.

Falkenmark and Folke [3] emphasize that the integration of social with ecological and hydrological elements is needed for sustainable catchment resilience. However, integration of various systems at the catchment scale sets up methodological challenges, especially integrating broader connected social and environmental processes. The resilience framework is an analytical framework that allows multiple interconnecting dimensions of catchments to be measured in an integrated fashion. An example of this, as mentioned above, is Walker et al’s work in the Goulburn Broken Catchment [38], where a resilience assessment was used to assess the sustainability of the basin. Here, the researchers focused on biophysical, economic and social elements of a region as components of a unified social ecological system. In such an assessment, whilst the focal scale is the region, there is an awareness of the scale below—farmers and householders—and the scale above—state and national legislation—that shapes the functioning of the catchment system at the regional scale. A resilience framing also includes the consideration of the possibility of regime shifts, and the identification of potential tipping points. Attempts to broaden the components of a system allows institutional and collective action elements to inform analysis of resilience.

The social elements of catchment resilience are diverse, and require a full range of observational, interactive and action-oriented research to both generate explanations of behavioral and institutional responses, but also to enact transformational change and study such processes from the inside out (known as action research). The diversity and richness of the social and integrative science methods, and the dimensions of the social dynamics they seek to explain are illustrated in Table 2. The commonalities of the three approaches, from observational to action research, include that they require significant resources, including time and labor, to ensure their rigor; that results are not always welcome to agencies and often challenge received wisdom concerning how societies and individuals act in apparently non-predictable manner; and that diversity of methods enhances rather than detracts from overall explanation. Hence, the use of qualitative observational methods, for example, often provide significant insight into causality and meaning that is lost in pattern-oriented quantitative descriptive analysis.

### Challenges to Inclusive Planning for Catchment Resilience

Resilience emerges from the interaction between different people as well as environmental processes: hence subjective desires of communities inform the definition of catchment resilience. As decision makers have to plan for multiple possible futures the use of novel participatory and deliberative processes, provide an opportunity for social learning, where new behaviors are acquired by observing, modelling and imitating others and learning takes place in a social context [58], amongst participants and improved fore-sight for landscape planning. Ultimately, methods that integrate aspects of learning seek to build resilience as well as measure and plan for it. A companion modelling approach is a multi-agent systems methodology that facilitates information sharing that can improve coordination among stakeholders for future collaboration and decision making. This research process begins with a problem definition developed by stakeholders and institutions and then the co-construction of models of the system with stakeholders. These system models are then tested using a number of simulations and subsequently actively run through participatory simulations with stakeholders exploring different possible scenarios e.g. the impacts of a flood event or new infrastructure on decision making. This iterative process of co-creation with stakeholders and running of scenarios means that the final models are more likely to often prominently reflect stakeholders needs. However, stakeholder and participatory approaches cannot be applied uncritically: it must be recognized that stakeholders often have diverging perspectives, interests and values, and different power and agency to shape and influence outcomes.

Participatory processes often require a significant time investment to get to know stakeholders, to agree on system dynamics, to run participatory simulations and then validate the final model. This means that, as well as resulting in catchment models, it potentially generates improved social networks for participants [59]. Serious games are a method that takes a futures approach – there are a broad collection of methods that allows an exploration of possible and preferable futures – these methods focus on desirable futures and generate insight into decisions that can be made in the present to improve management of social ecological systems with a particular future regime in mind [60].

Where the focus of enquiry is to examine the social impacts of different strategies for resilience, and to ask resilience for whom?, creative practice and participatory planning provide useful methodologies. Methods that enable input from local populations can introduce a focus on emotional aspects of catchment resilience into assessment [61]. This requires a move away from simply integrating social components into a model of catchment dynamics to an approach that puts normative aspects of fairness at the forefront of resilience assessments. This refocusing of resilience requires an investigation of the lived experience of people who live in, or are connected to, specific social ecological systems. For example, work using qualitative methods by Sims et al. [61] focused on a particular population group and studied the impact of flood events on carers, and their ability to continue their role of caring. The researchers used diary-based methodologies to understand how everyday practices of care are interrupted by floods. Such studies give rich insight into how social and environmental systems are related, and how nuanced resilience is when social processes are more fully integrated.

The question of resilience for whom can be extended beyond human interests to the interests of trees, rivers, and mountains. Indeed, in New Zealand in 2017 a river was given the same legal rights as a human. In giving these elements of a catchment voice and agency in research and planning processes the focus of resilience also shifts. Methods to extend the community of justice beyond humans require a certain degree of empathy with biotic and abiotic parts of social ecological systems. Here, existing methods such as scenarios and serious games could be adapted so that participants take on the identity of the non-human world. In this way marginal or sometimes unconsidered elements of a catchment can be placed more centrally in discussions of what constitutes and what supports catchment resilience.

## 6 Conclusions and Implications for Governance

Catchment resilience has significant potential as an organizing framework for the integrated and collaborative management of water resources in their social context. It is intuitively appealing, provides a way of understanding trade-offs and looking across specific sectoral or site-based interests, as well as being widely understood to be beneficial. Adopting catchment resilience also necessitates a recognition that elements outside the control of the system are likely to be important, ranging from global climate change, through to policy imperatives over which authorities at catchment level have little or no control. These include, for example, urban expansion, demands for infrastructure, or competing policy initiatives in other sectors.

A second implication for adopting an integrated catchment resilience approach is the need to recognize the benefits of diversity and capacities for self-organization and localized initiatives. The evidence from much social science is that the resilience of communities is enhanced by their perception of their own autonomy and agency, and that synergistic relationships with management authorities, rather than more combative ones, yield innovation and sustainability. Putting social processes central to catchment resilience assessments requires inclusive participatory processes from the beginning. In this way the definition of resilience can be co-determined by individuals, communities and public bodies. This process may then lead to suggestions of engagement with wider sets of people, systems or scales, for example engaging with businesses outside of the catchment whose supply chain is rooted in the catchment under consideration. By including such broader components into assessments the subsequent definition of what a resilient catchment is may change. In this way the methods used and the components analyzed as part of a resilient catchment can dynamically feed back into each other. Ultimately, this results in an ongoing negotiation of what catchment resilience is, which, whilst relatively resource intensive, promotes adaptiveness in responding to social and environmental change.

Managing catchment resilience is challenging for many reasons. First, a catchment may be shared amongst diverse institutions and communities who have different and sometimes opposing interests, as well established in existing watershed or catchment analysis [62]. Second, there are aspects of path dependency in how catchments are managed that make new practices difficult to implement. For example, when management has relied upon ‘hard engineering’ structures involving sunk costs, organizational practices and established knowledges and expertise, then it might be very difficult to make a shift towards new approaches, be they ecosystems-based approaches or more participatory management [63]. Furthermore, it must be acknowledged that participatory processes, no matter how innovative, may not be sufficient to overcome these challenges. The political dynamics within catchments suggest that there are significant constraints on making decisions that maximize diversity and re-distribute power: reputational risks to individuals and institutions in decision-making have been shown to be major sticking points for implementing catchment-oriented decision-making processes. For example, managing consultative processes and engagement with diverse stakeholders requires building trust and having transparent processes and clear roles and responsibilities. Consultation processes that lack legitimacy because they are not inclusive, or are seen as meaningless or empty gestures, and result in communities and stakeholders lose trust in management authorities.

This paper has highlighted that community resilience is an important dimension of catchment resilience. By using a social ecological systems lens, and emphasizing social processes and relations, some core challenges for catchment resilience are outlined, high-lighting the critical role of social relations and the need to understand and accommodate cross-scale social and environmental dynamics, and to design institutions to address their complexities.

## Figures and Tables

**Table 1:** Capacities supporting community resilience.

Community Resilience Capacity	Description	Explanation
Place attachment	The affective, cognitive and material relationship people have with place	Place attachment has been shown to enhance community resilience, but within limits as it may cause people to want to live in high-risk situations and make them less likely to accept new ideas and practices.
Leadership	People (leaders, entrepreneurs, champions), organisations, characteristics, roles and actions that affect outcomes	Leadership is important for knowledge and trust building and for effecting community action, but not sufficient for resilience.
Community networks	The bonding and bridging ties that enable people to act collectively	Community resilience is strengthened by access to diverse networks, providing essential support, help identify new opportunities and provide a focus for hope and optimism.
Community cohesion and efficacy	Community ability to act together and belief in one’s own ability to take action and manage situations	Supports community ability to act independently and to build resilience within the community itself.
Knowledge and learning	Individual and group capacity to respond to local needs and issues	Iterative, continuous and reflective learning supports community to respond to change, and enhance social memory.

Adapted from [23].

**Table 2:** Methods for measuring elements of resilience and action towards building resilience.

Scope	Indicative Methods	Examples
Observational methods, measurement and hypothesis testing	Testing hypotheses on existing spatial and social data on social and geographical distribution of risk and vulnerability Targeted surveys on elements of individual behaviour, perceptions and well-being. Observational ethnography, life history, and related intensive methods.	Geographic distribution of disadvantaged communities at risk across UK [12] Elicited narratives of flood recovery to uncover place and identity connections [47] Documentation of folk memories and narratives of living with flood risk [48] Multi-year repeated surveys of health consequences of floods and recovery [14] Storyboards, photovoice and visual methods to generate representations of place and meaning in flood recovery [49,50]
Interactive methods	Systems-oriented modelling of social-ecological dynamics and institutions Role playing and interactive games	Models of catchment systems used interactively with stakeholders to generate foresight on consequences of decisions [51] Development of role-playing games, used with stakeholders to generate foresight on consequence of decisions [52,53]
Action-oriented research and collaborative methods	Co-creation of knowledge for empowering community planning Creative practice and perspective taking activities Embedding scientific knowledge in self-organised community action and initiatives	Community-led generation of alternative future scenarios for catchments [54] Using theatre and performance art to stimulate engagement and empathy [55] Generation of artefacts, games and creative resources to stimulate engagement [53] Using local flood forums, community initiative and competency groups to generate local capacity for social learning and re-distributing expertise [56,57]

## Data Availability

No new data was produced or used for this paper.

## Box 1 Challenges of scale and fit in catchment resilience.

**Temporal scale**—trade-offs exist between maintaining or optimizing what exists now (infrastructure, economies, and values) and long-term options. This temporal dimension leads to moral hazard—short-term actions and interventions that compromise, limit, or trade-off actions in the future (e.g., maladaptation)

**Spatial scale**—actions in one place may have negative impacts elsewhere—either immediately downstream or in more remote places (e.g., teleconnections)

**Transboundary issues**—parts of the catchment have shared jurisdiction or cross boundaries

**Social–ecological interactions**—environmental variability interacts with complex social dynamics, such as place, identity, and human mobility

**Nonlinear dynamics**—social–ecological systems exhibit nonlinear or threshold responses to changes in climate variability and other stressors

**Cross-scale feedbacks**—complex interactions at different spatial or temporal scales generate thresholds and alternate stable states

**Institutional fit**—the scale of governance must be capable of responding to the scale of the policy problem and to the multiple scales of system processes, activities and uses.

Adapted from [37].
